# Material-Dependent Microhardness Response to Preheating in Nanoparticulate Composite Resins Cured with High-Intensity Light

**DOI:** 10.3390/dj13090403

**Published:** 2025-09-02

**Authors:** Jorge I. Fajardo, César A. Paltán, Ana Armas-Vega, Camila Campanella-Maldonado, Silvio Requena-Cisneros

**Affiliations:** 1 New Materials and Transformation Processes Research Group GiMaT, Universidad Politécnica Salesiana, Cuenca 010105, Ecuador; jfajardo@ups.edu.ec; 2 School of Dentistry, Universidad Central del Ecuador, Quito 170521, Ecuador; acarmas@uce.edu.ec (A.A.-V.); sccampanella@uce.edu.ec (C.C.-M.); sorequena@uce.edu.ec (S.R.-C.)

**Keywords:** polymerization, light curing, preheating, composite resins, Knoop microhardness

## Abstract

Background/Objectives: Composite resins are widely used in restorative dentistry due to their aesthetic properties and ease of handling. Preheating prior to light polymerization has been proposed to improve flowability, degree of conversion, and mechanical properties. This in vitro study aimed to evaluate the effect of preheating on the microhardness of three nanoparticulate composite resins—IPS Empress Direct (Ivoclar), Filtek Z350 XT (3M-ESPE), and Forma (Ultradent)—when cured with a high-power LED light. Methods: Sixty disc-shaped samples (*n* = 20 per material) were fabricated and divided into preheated and non-preheated groups. After polishing and 24 h storage in distilled water at 37 °C, samples were subjected to Knoop microhardness testing under a 300 g load for 15 s. Statistical analysis was conducted using R software. Results: Preheating produced a significant increase in surface microhardness for IPS Empress Direct (32.8%) and Filtek Z350 XT (5.8%) (*p* < 0.05 for both), whereas Forma showed no significant change. Conclusions: Under the conditions of this in vitro study, preheating can enhance the mechanical performance of specific composite resins by increasing microhardness; however, the effect is material-dependent.

## 1. Introduction

Over the last decade, the use of composite resins in daily clinical practice has increased significantly, driven by patients’ growing interest in aesthetic and functional restorations [1,2]. This widespread adoption has fueled research into material properties, focusing not only on advances in photoactivation techniques but also on structural changes in the organic matrix and the type, size, and content of filler particles [3]. These innovations have contributed to decreased polymerization shrinkage and, in some instances, increased viscosity, resulting in superior mechanical stability [4,5].

Despite these advances, challenges persist, particularly concerning adhesion and marginal sealing. The formation of interfacial spaces between the resin and the tooth can lead to microleakage and premature restoration failure. While incorporating flowable composites with less inorganic loading has been one strategy to mitigate this [6], preheating the material prior to polymerization has emerged as a promising technique to decrease viscosity and promote more effective marginal adaptation [7]. The theoretical basis of this technique is that an increase in temperature enhances monomer mobility, thereby increasing the monomer-to-polymer conversion [8]. This results in improved mechanical properties, such as higher surface hardness and flexural strength [9,10].

Furthermore, by reducing the proportion of unreacted monomers, preheating can minimize the risk of adverse reactions on pulp tissue and potential toxicity [11,12]. However, achieving complete polymer conversion remains a challenge for methacrylate-based materials, as conversions typically range from 50% to 75% [13,14]. At this point, factors such as the temperature reached, heating duration, lamp power, and the specific composition of the composite influence the polymerization efficiency [14]. For example, highly viscous resins may require longer light-curing times or higher lamp irradiance to achieve complete reaction. If not adequately controlled, this can lead to internal stresses and marginal cracks [4,15]. Preheating can mitigate such problems by favoring the initial flowability of the material and reducing the formation of voids at the cavity-resin interface [11,16].

The advent of high-power LED curing lamps has significantly advanced polymerization compared to traditional halogen units. These lamps generate higher irradiance levels, accelerating the degree of conversion in a shorter time [1]. However, increasing the speed of the polymer reaction carries a risk: if the material transitions too quickly from a viscous phase to a solid polymeric network, residual stresses intensify, potentially compromising bond integrity [4]. This underscores the critical importance of standardizing work protocols, such as preheating temperature, duration of light exposure, and lamp type, to maximize benefits and minimize potential adverse effects.

Within this scenario, nanoparticulated resins stand out for their aim to balance aesthetics, strength, and durability by including nano-sized filler particles [11]. These particles are more homogeneously distributed, which can improve mechanical properties and decrease the occurrence of surface defects. However, the response of these materials to preheating remains debated because each formulation presents a distinct organic matrix (Bis-GMA, UDMA, TEGDMA, or Bis-EMA) and a specific filler ratio [3]. This means that the conclusions drawn from one type of composite are not necessarily extrapolable to another, even if both contain nanometer-range filler particles.

On the other hand, microhardness has been established as one of the most widely used indicators to evaluate the final quality of a restoration [10]. Being directly associated with the ability to withstand masticatory loads and with microhardness, it is assumed that an increase in surface hardness reflects a higher degree of conversion and greater stability in the oral environment [9,17]. If preheating consistently contributes to increasing these values, it will represent a substantial advantage for daily clinical practice, potentially reducing early failures of restorations and the need for replacements or retreatments.

However, there is no agreement in the literature. Some authors report significant increases in microhardness after preheating. At the same time, other works observe differences that are not very relevant or depend on complementary factors, such as experimental design, light power, or material insertion technique [5,7]. Additionally, some research suggests that excessive heating may cause thermal damage to the pulp or volatilize specific components of the composite; therefore, the chosen temperature should be carefully adjusted [18,19].

Given these discrepancies and the need for more specific data on nanoparticle resins, this study aims to experimentally investigate the effect of preheating on the microhardness of three distinct nanoparticle composite resins: Forma, Filtek Z350, and IPS Empress Direct.

It is hypothesized that the microhardness of nanoparticulate composite resins increases with preheating, and that this effect is dependent on the specific brand of resin and composition.

## 2. Materials and Methods

We proposed an observational, comparative, and experimental study. The sample size used in this in vitro study was determined based on data from previously published research. Three direct-use composite resins of different compositions were used in vitro: Forma (Ultradent)—Ultradent Products, Inc., South Jordan, UT, USA; Filtek™ Z350 (3M-ESPE)—3M ESPE Dental Products (3M Company), St. Paul, MN, USA; (El IFU también lista 3M Deutschland GmbH, Neuss, Germany), and IPS Empress Direct (Ivoclar)—Ivoclar Vivadent AG, Schaan, Liechtenstein.

### 2.1. Sample Size

A total of *n* = 10 specimens per condition was selected based on precedent in comparable in vitro microhardness studies and on feasibility considerations for standardized specimen fabrication and testing [5]. No a priori power analysis was performed, because reliable estimates of variance and expected effect sizes were not available at the planning stage [17,18,19]. This approach is common in exploratory in vitro investigations of mechanical behavior, and we explicitly recommend conducting post hoc power analyses in future work once variance parameters are better established [5,17,18,19].

For the preheated group (G1), the composite resin material was preheated to 68 °C using a CalSet 3 temperature unit (AlDent) for 40 min at its highest setting, consistent with previous studies and literature review, which found that the 54 °C to 68 °C range is considered safe for the dental pulp and does not cause irritation [5]. Simultaneously, the metallic molds, clear glass cover slips, and plastic filling instrument were warmed to 37 °C before resin insertion. The non-preheated group (G2) was handled at room temperature. Importantly, the 40 min dwell at 68 °C was chosen to ensure thermal equilibration of syringes and ancillary instruments for research standardization; it does not represent typical chairside preheating times, which are generally shorter in clinical protocols.

Samples were prepared by placing the resin, using a composite spatula, onto a metallic matrix of 2 mm height and 6 mm diameter, previously positioned on a glass slab. Once the resin was inserted into the matrix, an object holder was placed to ensure a smooth external surface. Samples were then light cured with a Valo C high-power LED light (Ultradent) with a nominal intensity of 1200 mW/cm^2^. Curing was performed for 20 s, in a single exposure, at 1 mm between the lamp and the resin surface. A total of 20 samples were constructed for each resin material (*n* = 20 per material), with an equal distribution between the preheated and non-preheated groups for each resin type.

The samples were randomly assigned to their respective groups using a computer-generated randomization sequence. One operator was responsible for sample preparation, while a separate, blind researcher conducted the mechanical tests, unaware of the treatments applied to each sample.

The samples were polished (Femto 1500, provided by Pace Technologies, Tucson, AZ, USA) with a 0.3-micron colloidal alumina solution on both surfaces. Polishing was performed using a low-speed polishing system from 3M-ESPE, applied in sequence from coarse to fine grit for 5 s per disc. They were then stored for 24 h in dark bottles with distilled water at 37 °C to complete the polymerization, simulating the oral cavity.

### 2.2. Microhardness Test

Subsequently, the samples were subjected to microhardness evaluation. Knoop micro-hardness tests were performed on the top surfaces of each specimen using a microhardness tester (model G 20DT, supplied by Shimadzu, Austin, TX, USA), equipped with a Knoop diamond indenter. The tests were conducted with a dwell time of 15 s and a load of 300 g. Five indentations were performed per sample, spaced adequately to avoid interaction between them, ensuring a minimum distance of three times the diagonal indentation between each impression. An average Knoop Hardness Value (KHN) was calculated for each sample. Microhardness was determined by measuring the indentation diagonals produced.

A total of 60 samples were prepared (20 for each brand). The internal temperature of each preheated sample was verified. The Data obtained were processed with R v.4.3.3 software. The use of a high-power LED lamp for light curing and the standardized evaluation criteria aimed to isolate the real influence of preheating. By measuring the Knoop hardness and conducting statistical analysis of the results, we expected to determine the specific impact of this procedure on each of the selected materials, contributing to establishment of clinical guidelines that will enable the achievement of restorations with greater durability, better sealing, and a lower rate of residual monomers (Table 1).

## 3. Results

**Figure 1:**
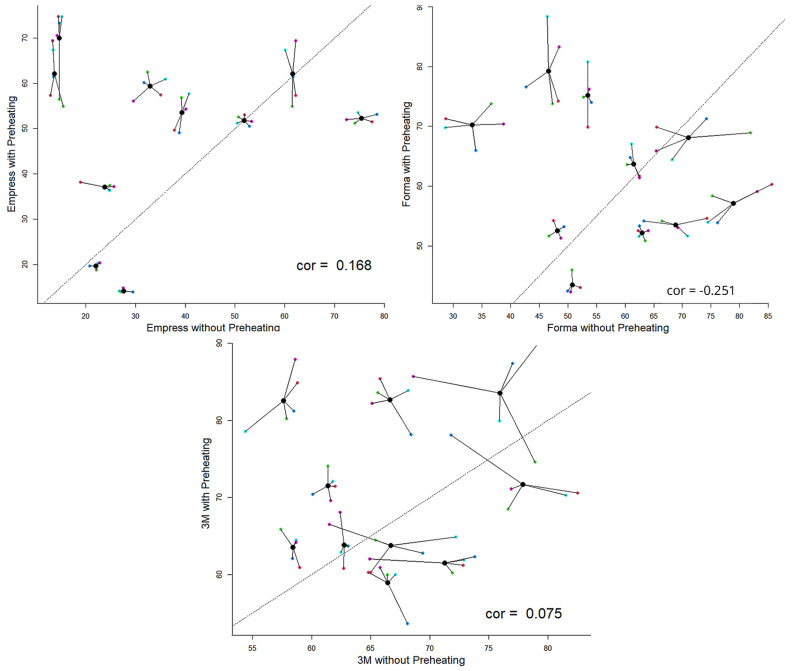
Contrast of values obtained with and without preheating per fragment by resin.

Considering the average Knoop microhardness value of each fragment obtained with and without preheating for each fragment and treating each fragment as a different sample, it was determined that the samples of Empress and 3M-ESPE achieved the highest hardness values with preheating. Simultaneously, Forma demonstrated consistent behavior, performed similarly both above and below the baseline. This indicates that Empress tends to increase in microhardness with preheating, as does 3M-ESPE, although they are not correlated. Additionally, Forma also exhibits this tendency, albeit with a decreasing trend (Figure 1).

The fundamental descriptive indicators varied according to the resin and condition preheating method. Table 2 presents the minimum, maximum, mode, median, and mean values, which differed across groups, with a tendency for values to be higher for certain resins (Table 2).

**Figure 2:**
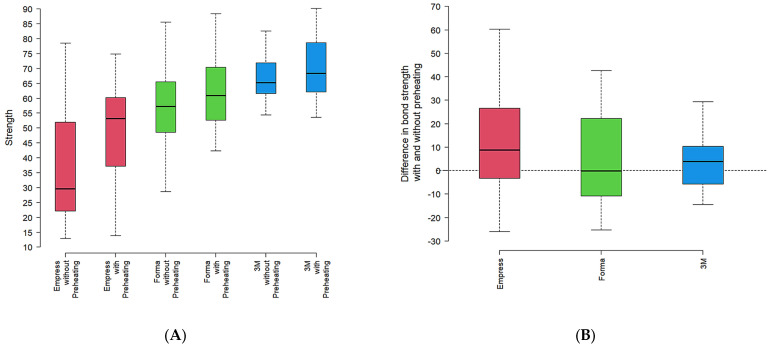
Box-and-whisker plots of Knoop microhardness (KHN) by resin and preheating condition (*n* = 20 per resin). Box-and-whisker plots of Knoop microhardness (KHN): (**A**) distribution by resin and preheating condition; (**B**) difference between with − without preheating by resin (ΔKHN = KHN_With − KHN_Without). *n* = 20 per resin.

In a complementary analysis, the data expressed in the box and whisker diagram (Figure 2A) illustrate the effect of data distribution according to the resin type and preheating condition.

Empress resin tended to show lower microhardness values, while Forma exhibited intermediate values with less variability. 3M-ESPE consistently displayed higher microhardness values and more homogeneous data. This trend was observed in both groups, with and without preheating conditions.

The difference in microhardness between preheating and no preheating was greater in the Empress resin (Figure 2B), with a difference in microhardness (KHN) between preheated and non-preheated groups for each resin. A similar pattern was seen in 3M-ESPE. Forma, however, presented an average difference close to zero, suggesting no significant change in microhardness with preheating (Figure 2B).

**Figure 3:**
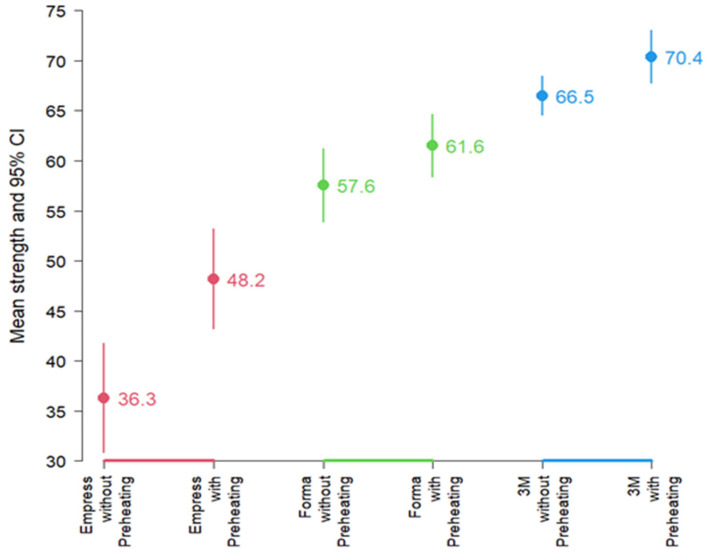
Mean Knoop microhardness and 95% Confidence Interval by resin and preheating condition.

Upon performing the normality test, all groups except the Empress group with preheating passed. Therefore, a Kruskal–Wallis test was performed, which yielded a *p* < 0.001, H = 24.6 (df: 5), indicating significant differences in microhardness among some of the groups. Paired *t* tests compared preheated vs. non-preheated within each resin; Figure 3 depicts mean KHN ± 95% CI by resin and condition., revealed that, at the average level, microhardness differed according to resin and activation. Resins with preheating generally showed higher average preheating generally showed higher average microhardness values than resins without preheating. In descending order of average microhardness, the 3M-ESPE resin exhibited higher values than Forma resin, which, in turn, showed higher average microhardness than Empress resin (Figure 3).

A hypothesis test for paired samples using the t-Student test was performed to compare preheated versus non-preheated groups for each resin. The results confirmed significant differences in microhardness between preheating and non-preheating for Empress (*p*: 0.001268) and 3M-ESPE (*p*: 0.02097). However, Forma (*p*: 0.1571) did not exhibit a significant difference at the average level.

Notably, the average microhardness of Empress resin increased by 32.8% with preheating. Forma showed a 6.9% increase, which is not statistically significant, and 3M-ESPE showed a 5.8% increase. This highlights that while Empress resin significantly improves its microhardness when preheated, Forma and 3M-ESPE did not show such a pronounced significant percentage increase with preheating despite showing higher values when preheated.

## 4. Discussion

The microhardness of both preheated and unheated 3M resins was superior to that of the other resins studied, which can be explained by the fact that the 3M-ESPE resin has a larger particle size compared to the Empress resin and a very similar size to the Forma resin [18]. However, the hardness of resins is dependent on their particle size, composition, and handling protocol, whether preheated or not [19]. The need to improve the mechanical properties of resins has led to the search for ways to achieve optimal levels of restorations, ensuring a proper marginal seal, decreasing polymerization shrinkage, and creating restorations that last over time [6], thereby justifying the execution of this study.

Surface hardness helps analyze the strength of the surface, with a direct relationship to the strength and mechanical properties of the material, which can be influenced by the clinician’s handling of the material [17]; on average, composite resins can achieve 50% to 70% conversion of monomers at room temperature [20], it is during this process that monomer conversion occurs as fast as light exposure is initiated, however as the reaction progresses, the viscosity of the resin composite increases through the formation and growth of polymer chains, resulting in a decrease in molecular motion [3], hence the requirement for the use of a lamp with adequate power to regulate this process.

In this conversion process, it is the increased viscosity of the resin that prevents the completion of the polymerization process because of the movement of the molecules in this vitrified state becomes limited [1]; preheated composites, on the other hand, exhibit a higher mobility of monomers, as a result of higher thermal energy, leading to lower viscosity and higher molecule movement which in turn allows a higher conversion of monomers to polymers [21], hence the new trend in restorative protocols is the use of preheated resins, thus, justifying the objective of this study.

The results obtained in this study indicate that resins with preheating show a higher microhardness than resins not subjected to preheating; however, the success is dependent on the size and composition of the resin filler [10], which would explain the results obtained where 3M Filtek Z350 XT resin has a combination of 20 nm silica filler, 4 to 11 nm zirconium filler and an average cluster particle size of 0.6 to 10 microns, the inorganic filler loading is approximately 78.5% by weight [22].

Empress Direct trade house resin has filler containing barium glass, ytterbium trifluoride, mixed oxides, and silicon dioxide. The total content of the inorganic filler is 75–79 wt%, and the particle size of the inorganic filler ranges from 40 nm to 3 µm, with an average particle size. The trade house resin form, which is present in the composition, contains zirconium and ytterbium trifluoride [23]. Over the years, there has been a continuous decrease in particle filler size from traditional materials to nanohybrids and nanoplates to obtain materials with improved mechanical properties and aesthetics [24]. These variations in average size, quantity, and volume create a large variety of composite categories, which allow choosing the right type of material for the treatment in case you want to preheat the resin as it is essential to use the right resin for the treatment to choose, so you need resins with lower viscosity and nanoparticulate.

A limitation in this study is the exclusive use of a 20 s photopolymerization time [25]. This restricts our ability to compare how varying exposure durations influence the mechanical properties of resins. Different polymerization times can significantly affect the degree of conversion, microhardness, and other physical properties, especially with high-power light-curing units. Therefore, future research should incorporate multiple exposure times to thoroughly assess their potential impact.

Additionally, it is important to consider whether the degree of monomer-to-polymer conversion in composite resins can continue to increase over time due to post-cure polymerization. Our mechanical tests were performed after a standardized period of 24 h, which may not fully reflect the long-term behavior of these materials. Further studies should assess the effects of aging, both in dry and moist conditions, over extended periods to better understand how time influences the stability and performance of both preheated and non-preheated resins.

It is important to note that the absence of artificial aging protocols, such as thermocycling between 5 °C and 55 °C, limits the extrapolation of results to long-term clinical conditions. Future studies should incorporate such aging methods to evaluate resin durability over time. The 40 min preheating protocol was selected based on the literature, as previous studies have shown this period allows the material to reach and maintain a uniform and stable temperature throughout its mass, ensuring consistent results [5]. The choice of the CalSet 3 and its use at its maximum temperature (68 °C) were based on manufacturer specifications, which designed the device to optimize the reduction in resin viscosity without compromising the integrity of its monomers and fillers. Therefore, our preheating schedule should be interpreted as a laboratory standardization strategy to achieve a uniform temperature profile across specimens. It is not intended to mirror chairside workflows, in which preheating durations are typically <10 min and depend on device capability and clinical constraints. In addition, the 40 min preheating dwell reflects laboratory standardization rather than clinically realistic timing, which should be kept in mind when extrapolating these results to chairside application.

As clinicians, in daily practice, we are often required to restore dental structures in which certain angles make the proper incorporation of the resin layers difficult. The possibility of preheating a resin opens new expectations for the use of resins in protocols where preheating increases the fluidity of the resin used, allowing for the most appropriate restoration of certain areas in the dental cavity, such as margins or undercuts, thereby ensuring the clinical success and longevity of the restoration performed. Knowing the composition of restorative material is a crucial element in achieving clinical success.

Preheating composite resins increase their temperature and reduce viscosity, resulting in greater fluidity. This improved flowability facilitates better adaptation of the material to the internal walls and margins of the cavity, especially in areas with complex anatomy or limited access. As a result, marginal sales have improved, which is critical for the long-term success of restorations. Improved sealing reduces the risk of microleakage, which is associated with postoperative sensitivity, bacterial infiltration, and the development of secondary caries. Therefore, incorporating preheating into restorative protocols can contribute to more predictable clinical outcomes and longer-lasting restorations. Additionally, the observed increase in microhardness due to preheating may also have a positive impact on the color stability of the material, an area that warrants further investigation in future studies [26]. The clinical application of these findings, particularly in terms of reducing marginal leakage and improving long-term restoration longevity, should be validated through in vivo studies to better assess their clinical significance.

## 5. Conclusions

Under the conditions of this in vitro study, preheating significantly increased the microhardness of two nanoparticulate composite resins (Empress and 3M-ESPE) when photoactivated with high-power LED light. No statistically significant effect was observed for the Forma resin, suggesting that the benefits of preheating may be material dependent. These results have important scientific and clinical implications. The observed increase in microhardness suggests a potential improvement in the mechanical performance and longevity of dental restorations made from Empress and 3M-ESPE resins. Specifically, in vivo studies are needed to validate these findings and assess the long-term clinical outcomes of preheating protocols. This finding is particularly relevant for high-stress areas in the oral cavity, where enhanced durability can reduce the risk of fractures and wear over time. Therefore, our study provides a scientifically backed rationale for the selective use of preheating as a strategy to improve the long-term clinical success of restorations with these specific materials.

## Figures and Tables

**Table 1:** Information on resins used in the experimental work.

Sample Color	Organic Matrix	Filler Type	Vol %	Particle Size Range	Expiry Date	Manufacturer
Forma Resin	A2 Enamel	Bis-GMA, TEGDMA, Bis-EMA, and UDMA	Colloidal silica, barium glass, strontium glass, zirconium dioxide	40–80%; 0.1 µm	2025	Ultradent
Filtek Z350 Resin (3M-ESPE)	A2 Enamel	Bis-GMA, UDMA, TEGDMA, and bis-EMA (6)	Silica, zirconia, zirconia/silica	72.5%; 4–11 nm (translucent shades)	2025	3M
Empress Direct Resin	A2 Enamel	Dimethacrylate	Barium glass, ytterbium trifluoride, mixed oxides, silicon dioxide, and copolymer	77.5–79% 40 nm–3 µm	2025	Ivoclar

**Table 2:** Basic descriptive statistics of Knoop microhardness (KHN) by resin and preheating condition method.

Resin	Preheating Condition	Min	Max	Mode	Median	Mean	SD	CV (%)	Lower CI	Upper CI
Empress	Non-preheated	12.9	78.5	23.5	38.9	36.3	19.7	54.2	30.9	41.7
	Preheated	13.9	74.8	55.4	55.0	48.2	18.1	37.5	43.2	53.2
Forma	Non-preheated	28.6	85.6	50.7	58.3	57.6	13.3	23.1	53.9	61.2
	Preheated	42.3	88.4	53.6	62.6	61.6	11.3	18.3	58.4	64.7
3M-ESPE	Non-preheated	54.4	82.5	63.0	65.9	66.5	7.0	10.5	64.6	68.4
	Preheated	53.6	90.1	63.1	68.9	70.4	9.5	13.5	67.7	73.0

## Data Availability

The data presented in this study is available on request from the corresponding author.
